# Associations Between Non-neurological Autoimmune Disorders and Psychosis: A Meta-analysis

**DOI:** 10.1016/j.biopsych.2018.06.016

**Published:** 2019-01-01

**Authors:** Alexis E. Cullen, Scarlett Holmes, Thomas A. Pollak, Graham Blackman, Dan W. Joyce, Matthew J. Kempton, Robin M. Murray, Philip McGuire, Valeria Mondelli

**Affiliations:** a Department of Psychosis Studies, South London and Maudsley NHS Foundation Trust, Institute of Psychiatry, Psychology & Neuroscience, King’s College London, London, United Kingdom; b Department of Psychological Medicine, South London and Maudsley NHS Foundation Trust, Institute of Psychiatry, Psychology & Neuroscience, King’s College London, London, United Kingdom; c National Institute for Health Research Maudsley Mental Health Biomedical Research Centre, South London and Maudsley NHS Foundation Trust, Institute of Psychiatry, Psychology & Neuroscience, King’s College London, London, United Kingdom

**Keywords:** Autoimmune, Epidemiology, Inflammation, Meta-analysis, Schizophrenia, Psychosis

## Abstract

**Background:**

A relationship between non-neurological autoimmune (NNAI) disorders and psychosis has been widely reported but not yet subjected to meta-analysis. We conducted the first meta-analysis examining the association between NNAI disorders and psychosis and investigated the effect of 1) temporality (as determined by study design), 2) psychiatric diagnosis, and 3) specific autoimmune disorders.

**Methods:**

Major databases were searched for articles published until April 2018; 31 studies, comprising data for >25 million individuals, were eligible. Using random-effects models, we examined the overall association between all NNAI disorders and psychosis; rheumatoid arthritis was examined separately given the well-established negative association with psychosis. Stratified analyses investigated the effect of temporality, psychiatric diagnosis, and specific NNAI disorders.

**Results:**

We observed a positive overall association between NNAI disorders and psychosis (odds ratio [OR] = 1.26; 95% confidence interval [CI], 1.12–1.41) that was consistent across study designs and psychiatric diagnoses; however, considerable heterogeneity was detected (*I*^2^ = 88.08). Patterns varied across individual NNAI disorders; associations were positive for pernicious anemia (OR = 1.91; 95% CI, 1.29–2.84), pemphigoid (OR = 1.90; 95% CI, 1.62–2.24), psoriasis (OR = 1.70; 95% CI, 1.51–1.91), celiac disease (OR = 1.53; 95% CI, 1.12–2.10), and Graves’ disease (OR = 1.33; 95% CI, 1.03–1.72) and negative for ankylosing spondylitis (OR = 0.72; 95% CI, 0.54–0.98) and rheumatoid arthritis (OR = 0.65; 95% CI, 0.50–0.84).

**Conclusions:**

While we observed a positive overall association between NNAI disorders and psychosis, this was not consistent across all NNAI disorders. Specific factors, including distinct inflammatory pathways, genetic influences, autoantibodies targeting brain proteins, and exposure to corticosteroid treatment, may therefore underlie this association.

SEE COMMENTARY ON PAGE 8

Findings from studies conducted over the past 6 decades have been used to support the claim that a relationship exists between non-neurological autoimmune (NNAI) disorders (i.e., autoimmune disorders largely affecting peripheral systems) and psychosis. In the 1950s, it was first observed that rheumatoid arthritis was less common among individuals with psychosis than in the general population 1, 2. Conversely, subsequent studies reported that other NNAI disorders, including celiac disease, systemic lupus erythematosus (SLE), and autoimmune thyroid disorders, were more prevalent among individuals with psychosis 3, 4, 5, 6. The most convincing evidence has come from several large-scale population studies that have demonstrated positive associations between schizophrenia and a range of autoimmune disorders 7, 8, 9. While there has been increased interest in this topic in light of evidence of altered immune system function in psychosis 10, 11, 12, the overall association between NNAI disorders and psychosis has yet to be investigated using meta-analytic techniques.

Quantifying the degree of association between NNAI disorders and psychosis and the extent to which this varies across study designs, specific psychiatric diagnosis, and individual NNAI disorders may help to elucidate the mechanisms that underlie any relationship and ultimately lead to the identification of more effective intervention strategies. Disentangling the temporal nature of this relationship is an important first step to determining whether these disorders simply co-occur more commonly than expected or whether NNAI disorders in fact increase the risk for psychosis. To this end, we conducted the first meta-analysis examining the association between NNAI disorders and psychosis. Only NNAI disorders were included owing to the well-established psychiatric manifestations of neurological autoimmune disorders. In the primary analysis, we examined effect sizes obtained from all studies regardless of design or diagnostic outcome; however, given the well-documented negative association between rheumatoid arthritis and psychosis, effect sizes for this disorder were examined in isolation. Stratified analyses were conducted to further examine the extent to which 1) temporality (as determined by study design), 2) specific psychiatric diagnoses (schizophrenia vs. more broadly defined psychosis vs. nonschizophrenia psychosis), and 3) specific NNAI disorders influenced the magnitude and consistency of any effect.

## Methods and Materials

### Search Strategy

As far as possible, the search strategy was conducted in accordance with the Meta-analysis Of Observational Studies in Epidemiology guidelines (13), as detailed in Supplemental Table S1. In brief, PubMed, PsycINFO, EMBASE, WorldCat dissertations and theses, and Global Health databases were searched for all articles published until April 2018; reference lists of review articles were manually searched. Search terms included psychosis or schizophrenia or nonaffective psychosis or clinical psychotic symptoms combined with autoimmune disorders or autoimmune diseases or XXX, the last-mentioned representing 37 individual NNAI disorders (Supplemental Table S2). Autoimmune disorders included in the search strategy were selected a priori from the American Autoimmune and Related Diseases Association (14); all disorders were cross-checked against known neurological disorders, as listed by the American Academy of Neurology (15), and only disorders that did not appear here were deemed eligible for inclusion. Full texts of articles were retrieved as necessary, and study authors were contacted where these were unavailable.

### Selection Criteria

Detailed inclusion and exclusion criteria are provided in Supplemental Table S3. After a preliminary screen (title and abstract) to exclude studies that clearly did not meet eligibility criteria, two of the authors (AEC and SH) reviewed the full text of all potentially eligible studies to determine inclusion. Disagreements were resolved by discussion with all study authors.

### Data Extraction

Two researchers (AEC and SH) independently extracted information on year of publication, psychiatric diagnosis, autoimmune disorders, country, study design, data source, sample size, mean or median age of sample, participant sex, matching factors, and outcome measure (prevalence or incidence). To examine the effect of temporality, all eligible studies were categorized as follows: 1) studies examining the comorbidity of NNAI disorders and psychosis (type A), 2) studies in which the autoimmune disorder preceded the onset or measurement of the psychotic disorder (type B), and 3) studies in which the psychotic disorder preceded the onset or measurement of the autoimmune disorder (type C).

We included studies providing prevalence or incidence data on the basis that psychotic and autoimmune disorders are both rare, thus, odds and risk ratios are likely comparable (16). To pool data across studies reporting different effect size measures, we extracted raw data from all eligible studies as follows: the number of individuals with both psychosis and an autoimmune disorder (a), the number of individuals with psychosis who did not have an autoimmune disorder (b), the number of individuals without psychosis who had an autoimmune disorder (c), and the number of individuals who had neither psychosis nor an autoimmune disorder (d). Extracted data were used to compute odds ratios (ORs) [(a/c)/(b/d)] with 95% confidence intervals (CIs); a continuity correction of 0.5 was applied to cells with zero counts (17). Thus, our pooled effect sizes represent raw (unadjusted) associations.

Where the stated aim of the study was to examine autoimmune disorders, and efforts were made by the authors to distinguish these from similar disorders with other (nonautoimmune) causes, we extracted data for all disorders classified as autoimmune by the authors (excepting those with a neurological basis); this included various forms of anemia 8, 9, 18. For studies reporting data for both schizophrenia and more broadly defined psychosis (where the latter included schizophrenia), we subtracted schizophrenia cases from the total number of psychosis cases to obtain mutually exclusive groups. Where studies examined multiple NNAI disorders and/or more than one psychiatric outcome, data were extracted for each NNAI disorder and/or psychiatric outcome separately such that a single study could provide multiple effect sizes. Authors were contacted when these data were not reported in the publication, with data provided for some studies 4, 18, 19, 20, 21, 22, but not others 7, 23, 24, 25.

Eligible studies were assessed for quality by the first author (AEC) using a modified version of the Newcastle-Ottawa Scale (26). The Newcastle-Ottawa Scale provides separate assessment criteria for cross-sectional, case-control, and cohort studies, covering three methodological domains (selection criteria, comparability, and measurement of exposure and/or outcome). Scoring criteria were amended such that the maximum score available for each study design was 8 (Supplemental Table S4).

### Statistical Analyses

Meta-analyses were conducted using Comprehensive Meta Analysis Software Version 3.0 (Biostat, Englewood, NJ). Given that the studies varied with respect to design and specific autoimmune disorders examined, we anticipated that the true effect would vary across studies; all analyses were therefore conducted using a random-effects model with inverse weighting applied (27). Stratified analyses were used to explore potential mechanisms and sources of heterogeneity. We first examined the overall association between NNAI disorders and psychosis, excluding effect sizes pertaining to rheumatoid arthritis. Next, we explored the effect of temporality by conducting analyses for each of the separate study design types defined above (A, B, and C). In the third step, we stratified by psychiatric diagnosis to determine whether the association with autoimmune disorders varied according to whether the outcome was schizophrenia, more broadly defined psychosis (including schizophrenia), or nonschizophrenia psychosis. Finally, meta-analyses were conducted for individual autoimmune disorders where more than three effect sizes were available. Statistical significance was set at *p* < .05 (two-tailed) for all analyses. Heterogeneity was assessed via the Cochran *Q* statistic (to identify statistically significant heterogeneity) and the *I*^2^ statistic (to estimate the percentage of the variability in ORs owing to heterogeneity) where classification of the latter as likely unimportant (0–40%), moderate (30%–60%), substantial (50%–90%), or considerable (75%–100%) was dependent on the magnitude and/or direction of effects and statistical significance of heterogeneity (28). Given the problems associated with applying statistical tests to assess small sample bias (publication bias) in meta-analyses with binary outcomes, particularly when significant heterogeneity is present (29), small sample bias was assessed visually by means of a funnel plot for analyses with 10 or more effect sizes.

## Results

### Search Results

**Figure 1:**
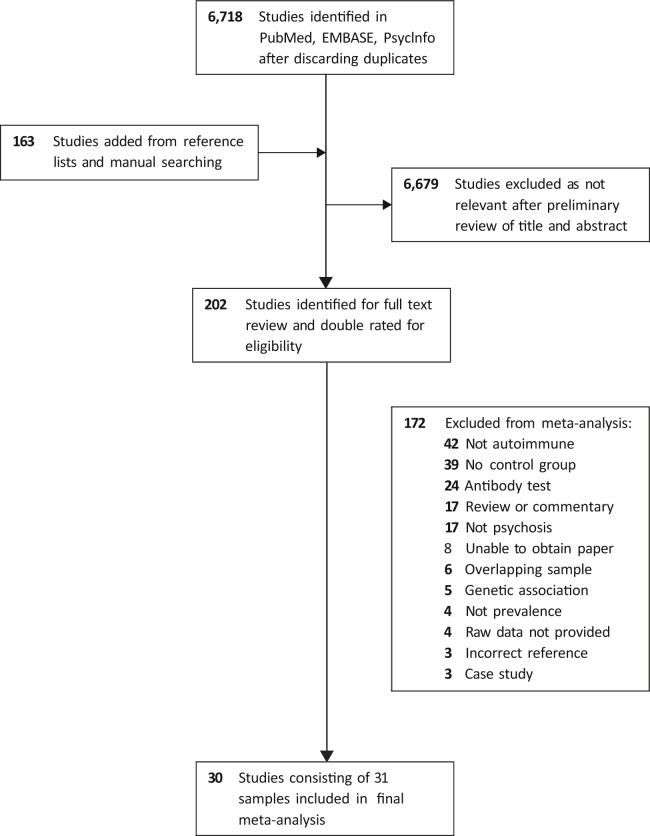
Search process. Overview of the review process and reasons for exclusion.

Thirty publications 4, 8, 9, 18, 19, 20, 21, 22, 30, 31, 32, 33, 34, 35, 36, 37, 38, 39, 40, 41, 42, 43, 44, 45, 46, 47, 48, 49, 50, 51 yielding 31 studies and 107 effect sizes met inclusion criteria (Figure 1). This included one study originally published as a conference abstract (later withdrawn as the author was unable to attend) (18), but for which statistical outputs for all analyses were kindly provided by the study author. Study details are provided in Table 1; the total number of individuals included across all studies was 25,041,429. Quality rating scores ranged from 2 to 8 (mean ± SD: 5.06 ± 1.29).

**Table 1:** Details and Quality Rating for Studies Meeting Inclusion Criteria

Reference	Type^a^	Psychiatric Diagnosis (Measure)	Autoimmune Disorder (Measure)	Country	Design (E/O)^b^	Data Source	Sample	Age, Years^c^	Male, %	Matching Factors	Outcome Measure	Quality Score (Maximum 8)
Allebeck *et al.*, 1985 (30)	C	Schizophrenia and affective psychosis (ICD-7)	Rheumatoid arthritis (ICD-7)	Sweden	Cohort (PSY/NNAI)	County inpatient register	PSY (*n* = 1811) Cont (*n* = 16,617)	NS	PSY: 38.9 Cont: 44.2	None	Incidence	5
Butwicka *et al.*, 2015 (31)	B	Psychosis (ICD-8, ICD-9, ICD-10)	Childhood-onset type 1 diabetes (ICD-8, ICD-9)	Sweden	Cohort (NNAI/PSY)	National diabetes register	NNAI (*n* = 17,122) Cont (*n* = 1,696,611)	NNAI: 9.3 Cont: NS	NNAI: 54.1 Cont: 53.9	Age, sex, country of birth	Incidence	6
Butwicka *et al.*, 2017 (22)	B/C	Psychosis (ICD-8, ICD-9, ICD-10)	Childhood-onset celiac disease (biopsy)	Sweden	A: Cohort (NNAI/PSY) B: Case control (PSY/NNAI)	Histological data and national patient register	NNAI (*n* = 10,903) Cont (*n* = 1,042,072)	NS	NNAI: 37.3 Cont: 38.8	Age, sex, country of birth	Incidence/prevalence	6/6
Chen *et al.*, 2011 (32)	C	Schizophrenia (ICD-9)	Pemphigoid (ICD-9)	Taiwan	Case-control (PSY/NNAI)	National health insurance database	NNAI (*n* = 3485) Cont (*n* = 17,425)	NNAI: 74.0 Cont: 74.0	NNAI: 54.8 Cont: 54.8	Age, sex	Prevalence	6
Chen *et al.*, 2012 (8)	A	Schizophrenia (ICD-9)	All NNAI (*n* = 25) (ICD-9)	Taiwan	Case-control (NNAI/PSY)	National health insurance database	PSY (*n* = 10,811) Cont (*n* = 108,110)	NS	PSY: 54.9 Cont: 49.5	Age	Prevalence	5
Chu *et al.*, 2012 (33)	A	Schizophrenia (ICD-9)	Alopecia areata (ICD-9)	Taiwan	Case-control (PSY/NNAI)	National health insurance database	NNAI (*n* = 5117) Cont (*n* = 20,468)	NS	NNAI: 50.8 Cont: 50.8	Age, sex	Prevalence	5
Cremaschi *et al.*, 2017 (34)	A	Schizophrenia (NS)	All NNAI (*n* = 6) (NS)	Sweden	Case-control (NNAI/PSY)	Hospital discharge register and patient interview	PSY (*n* = 5278) Cont (*n* = 6485)	PSY: 53.9 Cont: 56.3	PSY: 59.7 Cont: 51.2	None	Prevalence	3
Eaton *et al.*, 2006 (9)	B	Schizophrenia (ICD-8, ICD-10)	All NNAI (*n* = 24) (ICD-8, ICD-10)	Denmark	Case-control (NNAI/PSY)	National psychiatric and patient register	PSY (*n* = 7704) Cont (*n* = 192,590)	NS	PSY: 66.0 Cont: 66.0	Age, sex	Prevalence	5
Forsti *et al.*, 2016 (35)	B/C	Schizophrenia (ICD-9, ICD-10)	Pemphigoid (ICD-9, ICD-10)	Finland	A: Cohort (NNAI/PSY) B: Case-control (PSY/NNAI)	National register for health care	NNAI (*n* = 4524) Cont (*n* = 66,138)	NNAI: 77.0 Cont: 73.0	NNAI: 40.0 Cont: 45.0	None	Incidence/prevalence	5/5
Guerin *et al.*, 2012 (19)	A	Psychosis (ICD-9)	Psoriasis (ICD-9)	USA	Case-control (PSY/NNAI)	National health insurance database	NNAI (*n* = 106,128) Cont (*n* = 106,128)	NNAI: 52.1 Cont: 52.1	NNAI: 48.5 Cont: 48.5	Age, sex	Prevalence	5
Huilaja *et al.*, 2018 (36)	A	Psychosis (ICD-9, ICD-10)	Dermatological NNAI (*n* = 2) (ICD-9, ICD-10)	Finland	Case-control (PSY/NNAI)	National register for health care	NNAI (*n* = 21,690) Cont (*n* = 17,488)	NNAI: 41.2 Cont: 40.5	NNAI: 41.5 Cont: 41.3	Age, sex	Prevalence	5
Hutchinson *et al.*, 1996 (4)	A	Psychosis (DSM-III)	SLE (NS)	Trinidad	Case-control (PSY/NNAI)	Outpatient sample	NNAI (*n* = 45) Cont (*n* = 48)	NS	NS	None	Prevalence	—
Juvonen *et al.*, 2007 (37)	B	Schizophrenia (ICD-8, DSM-III-R)	Type 1 diabetes (NS)	Finland	Cohort (NNAI/PSY)	National population register	Entire population (*N* = 896,175)	NS	NS	None	Incidence	5
Kridin *et al.*, 2017 (38)	A	Schizophrenia (NS)	Pemphigoid (NS)	Israel	Case-control (PSY/NNAI)	Health services database	NNAI (*n* = 1985) Cont (*n* = 9874)	NNAI: 72.1 Cont: 72.1	NNAI: 40.2 Cont: 40.1	Age, sex, ethnicity	Prevalence	3
Lauerma *et al.*, 1998 (39)	B	Schizophrenia (DSM-III-R)	Rheumatoid arthritis (NS)	Finland	Cohort (NNAI/PSY)	National hospital discharge database	NNAI (*n* = 5626) Cont (*n* = 5330)	NS	NS	None	Incidence	4
Lauerma *et al.*, 1998 (39)	A	Schizophrenia (DSM-III-R)	Rheumatoid arthritis (NS)	Northern Finland	Cross-sectional (NNAI/PSY)	National hospital discharge database	PSY (*n* = 76) Cont (*n* = 10,503)	NS	NS	None	Prevalence	3
Ludvigsson *et al.*, 2007 (40)	B	Schizophrenia and nonaffective psychosis (ICD-8, ICD-9, ICD-10)	Celiac disease (ICD-7, ICD-8, ICD-9, ICD-10)	Sweden	Cohort (NNAI/PSY)	National inpatient register	NNAI (*n* = 14,003) Cont (*n* = 68,125)	NS	PSY: 41.1 Cont: 41.0	Age, sex, area of residence	Incidence	7
Marrie *et al.*, 2018 (21)	A	Schizophrenia (ICD-9, ICD-10)	Rheumatoid arthritis (ICD-9, ICD-10)	Canada	Case-control (PSY/NNAI)	National health database	NNAI (*n* = 6350) Cont (*n* = 33,584)	NNAI: 53.7 Cont: 53.7	NNAI: 27.8 Cont: 27.8	Age, sex, geographic region	Prevalence	8
Mors *et al.*, 1999 (41)	B	Schizophrenia (ICD-8)	Rheumatoid arthritis (adult and juvenile) (ICD-8)	Denmark	Case-control (NNAI/PSY)	National psychiatric and patient register	PSY (*n* = 20,495) Cont (*n* = 204,912)	NS	PSY: 57.6 Cont: 57.6	Age, sex	Prevalence	5
Petrak *et al.*, 2003 (42)	C	Possible psychosis (DSM-IV)	Type 1 diabetes (NS)	Germany	Case-control (PSY/NNAI)	Inpatient sample (cases) and general population (controls)	NNAI (*n* = 313) Cont (*n* = 2046)	NNAI: 28.3 Cont: 30.2	NNAI: 62.3 Cont: 50.8	None	Prevalence	4
Rajkumar *et al.*, 2017 (43)	A	Schizophrenia (ICD-8, ICD-10)	Type 1 diabetes (ICD-8, ICD-10)	Denmark	Cohort (PSY/NNAI)	National patient registers and prescription registry	PSY (*n* = 8945) Cont (*n* = 2,727,565)	NS	PSY: 59.7 Cont: 50.8	None	Incidence	5
Rothermich and Philips, 1963 (44)	A	Psychosis (NS)	Rheumatoid arthritis (NS)	USA	Case-control (NNAI/PSY)	Hospital records with NNAI screening	PSY (*n* = 16,000) Cont (*n* = 4040)	NS	NS	None	Prevalence	2
Schmitt and Ford, 2010 (45)	A	Schizophrenia (ICD-10)	Psoriasis (ICD-10)	Germany	Case-control (PSY/NNAI)	Outpatient records database	NNAI (*n* = 3147) Cont (*n* = 3147)	NNAI: 57.1 Cont: 57.1	NNAI: 44.7 Cont: 44.7	Age, sex	Prevalence	6
Sellgren *et al.*, 2014 (20)	C	Schizophrenia (ICD-8, ICD-9, ICD-10)	Rheumatoid arthritis and ankylosing spondylitis (ICD-8, ICD-9, ICD-10)	Sweden	Cohort (PSY/NNAI)	National population register	Entire population (*N* = 6,413,683)	PSY: 44.0 Cont: NS	PSY: 59.0 Cont: NS	None	Incidence	6
Shen *et al.*, 2016 (46)	B	Schizophrenia (ICD-9)	Ankylosing spondylitis (ICD-9)	Taiwan	Cohort (NNAI/PSY)	National health insurance database	NNAI (*n* = 2331) Cont (*n* = 9324)	NNAI: 36.5 Cont: 36.5	NNAI: 64.9 Cont: 63.9	Age, sex	Incidence	7
Sundquist *et al.*, 2008 (47)	B	Schizophrenia and psychosis (ICD-8, ICD-9, ICD-10)	Rheumatic NNAI (*n* = 3) (ICD-8, ICD-9, ICD-10)	Sweden	Cohort (NNAI/PSY)	National hospital discharge register	Entire population (*N* = 8,142,857^*d*^)	NS	NS	None	Incidence	6
Tiosano *et al.*, 2017 (48)	A	Schizophrenia (NS)	SLE (NS)	Israel	Case-control (PSY/NNAI)	Health services database	NNAI (*n* = 5018) Cont (*n* = 25,090)	NNAI: 50.2 Cont: 50.2	NNAI: 18.0 Cont: 18.0	Age, sex	Prevalence	3
Tu *et al.*, 2017 (49)	A	Schizophrenia (ICD-9)	Psoriasis (ICD-9)	Taiwan	Cross-sectional (NNAI/PSY)	National health insurance database	NNAI (*n* = 10,796) Cont (*n* = 767,327)	NNAI: 50.5 Cont: 45.9	NNAI: 55.2 Cont: 48.4	None	Prevalence	6
Weber *et al.*, 2013 (18)	A	Schizophrenia (ICD-9)	All NNAI (*n* = 9) (ICD-9)	USA	Cross-sectional (NNAI/PSY)	Hospital discharge database	Entire sample (*N* = 2,875,233)	NS	NS	None	Prevalence	5
West *et al.*, 2006 (50)	A	Schizophrenia (NS)	Gastrointestinal NNAI (*n* = 3) (NS)	UK	Case-control (PSY/NNAI)	Primary care database	NNAI (*n* = 18,994) Cont (*n* = 95,052)	NS	NS	Age, sex, GP, FU time	Prevalence	5
Yu *et al.*, 2017 (51)	C	Schizophrenia (ICD-9)	Psoriasis (ICD-9)	Taiwan	Cohort (PSY/NNAI)	National health insurance database	PSY (*n* = 4980) Cont (*n* = 19,920)	PSY: 46.5 Cont: 46.6	PSY: 50.7 Cont: 50.7	Age, sex	Incidence	5

Cont, control; FU, follow-up; GP, general practitioner; ICD, International Classification of Diseases of the World Health Organization; NNAI, non-neurological autoimmune (disorder); NS, not specified; PSY, psychiatric disorder; SLE, systemic lupus erythematosus.

a Type: A, comorbidity of schizophrenia/psychosis and autoimmune; B, autoimmune precedes schizophrenia/psychosis; C, schizophrenia/psychosis precedes autoimmune.

b Study design: E, exposure; O, outcome.

c Age: mean or median.

### Global Association Between Autoimmune Disorders and Psychosis

**Figure 2:**
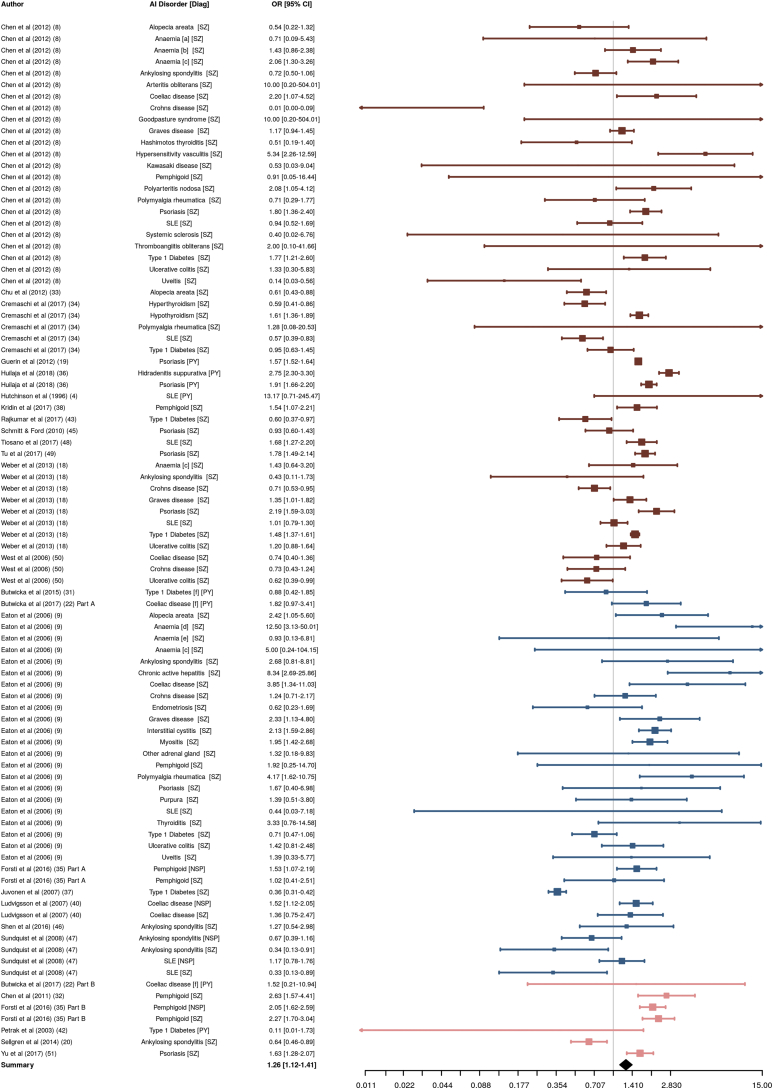
Results of overall meta-analysis for all non-neurological autoimmune disorders (except rheumatoid arthritis) and psychosis. Marker and line colors indicate study design: type A (red), type B (blue), type C (pink). AI, autoimmune; CI, confidence interval; Diag, psychiatric diagnosis; HSV, hypersensitivity vasculitis; NSP, nonschizophrenia psychosis; OR, odds ratio; PY, psychosis; SLE, systemic lupus erythematosus; SZ, schizophrenia. ^a^Autoimmune hemolytic type. ^b^Hereditary hemolytic type. ^c^Pernicious type. ^d^Acquired hemolytic. ^e^Other hereditary hemolytic type. ^f^Childhood-onset.

An analysis was first conducted to test the global association between all NNAI disorders, excluding rheumatoid arthritis, and psychosis. As shown in Figure 2, a significant positive association was observed (OR = 1.26; 95% CI, 1.12–1.41); however, considerable between-study heterogeneity was detected (Table 2). The funnel plot (Supplemental Figure S1A) showed no evidence of asymmetry.

**Table 2:** Results of Meta-analyses Examining Associations Between Non-neurological Autoimmune Disorders and Psychosis

Analysis	Number of Studies (Type)	Number of Effect Sizes (Diagnosis)	*N* (PSY/NNAI)	OR (95% CI)	*p*	*Q* (*p*)	*I*^2^ (95% CI)
Overall^a^	27 (A = 13; B = 8; C = 6)	90 (SZ = 77; BDP = 8; NSP = 5)	641,613/540,349	1.26 (1.12–1.41)^b^	< .001^b^	< .001^b^	88.08 (85.94–89.89)
Temporal Relationship^a^							
Comorbidity (A)	7 (A = 13)	49 (SZ = 45; BDP = 4)	410,627/328,199	1.20 (1.06–1.35)^b^	.003^b^	< .001^b^	84.80 (80.67–88.04)
NNAI precedes PSY (B)	6 (B = 8)	34 (SZ = 28; BDP = 2; NSP = 4)	193,594/176,578	1.43 (1.04–1.95)^b^	.03^b^	< .001^b^	88.58 (85.10–91.25)
PSY precedes NNAI (C)	3 (C = 6)	7 (SZ = 4; BDP = 2; NSP = 1)	37,392/35,572	1.55 (1.01–2.38)^b^	.046^b^	< .001^b^	87.14 (75.77–93.18)
Psychiatric Diagnosis^a^							
Schizophrenia	20 (A = 10; B = 6; C = 4)	77 (SZ = 77)	615,498/290,506	1.21 (1.04–1.40)^b^	.01^b^	< .001^b^	87.08 (84.50–89.23)
Psychosis (broadly defined)	7 (A = 3; B = 2; C = 2)	8 (BDP = 8)	14,241/167,104	1.81 (1.39–2.37)^b^	< .001^b^	< .001^b^	85.60 (73.58–92.16)
Nonschizophrenia psychosis	4 (B = 3; C = 1)	5 (NSP = 5)	11,874/82,739	1.38 (1.01–1.88)^b^	.046^b^	.003^b^	75.34 (39.36–89.97)
Autoimmune Disorder							
Alopecia areata	3 (A = 2; B = 1)	3 (SZ = 3)	18,777/5283	0.90 (0.38–2.10)	.80	.010^b^	78.26 (29.97–93.25)
Anemia (pernicious)	3 (A = 2; B = 1)	3 (SZ = 3)	32,239/1009	1.91 (1.29–2.84)^b^	.001^b^	.61	0.00 (0.00–93.12)
Ankylosing spondylitis	6 (A = 2; B = 3; C = 1)	7 (SZ = 6; NSP = 1)	73,967/63,198	0.72 (0.54–0.98)^b^	.04^b^	.14	37.54 (0.00–73.70)
Celiac disease	6 (A = 2; B = 3; C = 1)	7 (SZ = 4; BDP = 2; NSP = 1)	19,507/54,624	1.53 (1.12–2.10)^b^	.008^b^	.131	39.08 (0.00–74.38)
Crohn’s disease	4 (A = 3; B = 1)	4 (SZ = 4)	32,364/20,907	0.67 (0.34–1.30)	.23	.002^b^	79.97 (46.98–92.44)
Graves’ disease	3 (A = 2; B = 1)	3 (SZ = 3)	32,239/7799	1.33 (1.03–1.72)^b^	.03^b^	.18	41.19 (0.00–82.07)
Pemphigoid	6 (A = 2; B = 2; C = 2)	8 (SZ = 6; NSP = 2)	20,232/23,585	1.90 (1.62–2.24)^b^	< .001^b^	.322	13.81 (0.00–56.59)
Polymyalgia rheumatica	3 (A = 2; B = 1)	3 (SZ = 3)	23,354/112	1.63 (0.41–6.48)	.49	.030	71.35 (2.74–91.56)
Psoriasis	8 (A = 6; B = 1; C = 1)	8 (SZ = 6; BDP = 2)	54,578/141,673	1.70 (1.51–1.91)^b^	< .001^b^	.010^b^	61.94 (17.82–82.38)
Rheumatoid arthritis	12 (A = 6; B = 4; C = 2)	17 (SZ = 14; BDP = 1; NSP = 2)	244,320/125,090	0.65 (0.50–0.84)^b^	.001^b^	< .001^b^	79.28 (67.52–86.79)
SLE	7 (A = 5; B = 2)	8 (SZ = 6; BDP = 1; NSP = 1)	48,140/66,545	0.95 (0.65–1.39)	.80	< .001^b^	76.91 (54.10–88.39)
Type 1 diabetes	8 (A = 4; B = 3; C = 1)	8 (SZ = 6; BDP = 2)	47,208/132,921	0.79 (0.43–1.46)	.46	< .001^b^	97.31 (96.10–98.14)
Ulcerative colitis	4 (A = 3; B = 1)	4 (SZ = 4)	32,420/15,526	1.04 (0.69–1.56)	.86	.08	56.20 (0.00–85.48)

BDP, broadly defined psychosis; CI, confidence interval; NNAI, non-neurological autoimmune (disorder); NSP, nonschizophrenia psychosis; OR, odds ratio; PSY, psychiatric disorder; SLE, systemic lupus erythematosus; SZ, schizophrenia.

a Effect sizes for rheumatoid arthritis excluded from analyses. Temporal relationship group: A, comorbidity of schizophrenia/psychosis and autoimmune; B, autoimmune diagnosis precedes schizophrenia/psychosis; C, schizophrenia/psychosis diagnosis precedes autoimmune.

b Statistical significance at .05 level (two-tailed).

### Stratification by Temporal Relationship

The temporal association between NNAI disorders (excluding rheumatoid arthritis) and psychosis was investigated by conducting separate meta-analyses for studies examining the comorbidity of these disorders (type A), studies in which the autoimmune disorder preceded psychosis (type B), and studies in which psychosis preceded the autoimmune disorder (type C). Significant positive associations were observed for all three study types: A (OR = 1.20; 95% CI, 1.06–1.35), B (OR = 1.43; 95% CI, 1.04–1.95), C (OR = 1.55; 95% CI, 1.01–2.38). However, there was considerable heterogeneity between studies within each type (Table 2).

### Stratification by Psychiatric Outcome

Of the 90 effect sizes included in the main analysis (i.e., excluding rheumatoid arthritis), 77 examined schizophrenia, 8 examined a more broadly defined psychosis outcome that included schizophrenia, and 5 examined nonschizophrenia psychosis. As shown in Table 2, ORs were positive and statistically significant for all three psychiatric diagnostic outcomes: schizophrenia (OR = 1.21; 95% CI, 1.04–1.40), broadly defined psychosis (OR = 1.81; 95% CI, 1.39–2.37), nonschizophrenia psychosis (OR = 1.38; 95% CI, 1.01–1.88). However, heterogeneity was considerable for all three outcomes.

### Stratification by Autoimmune Disorder

**Figure 3:**
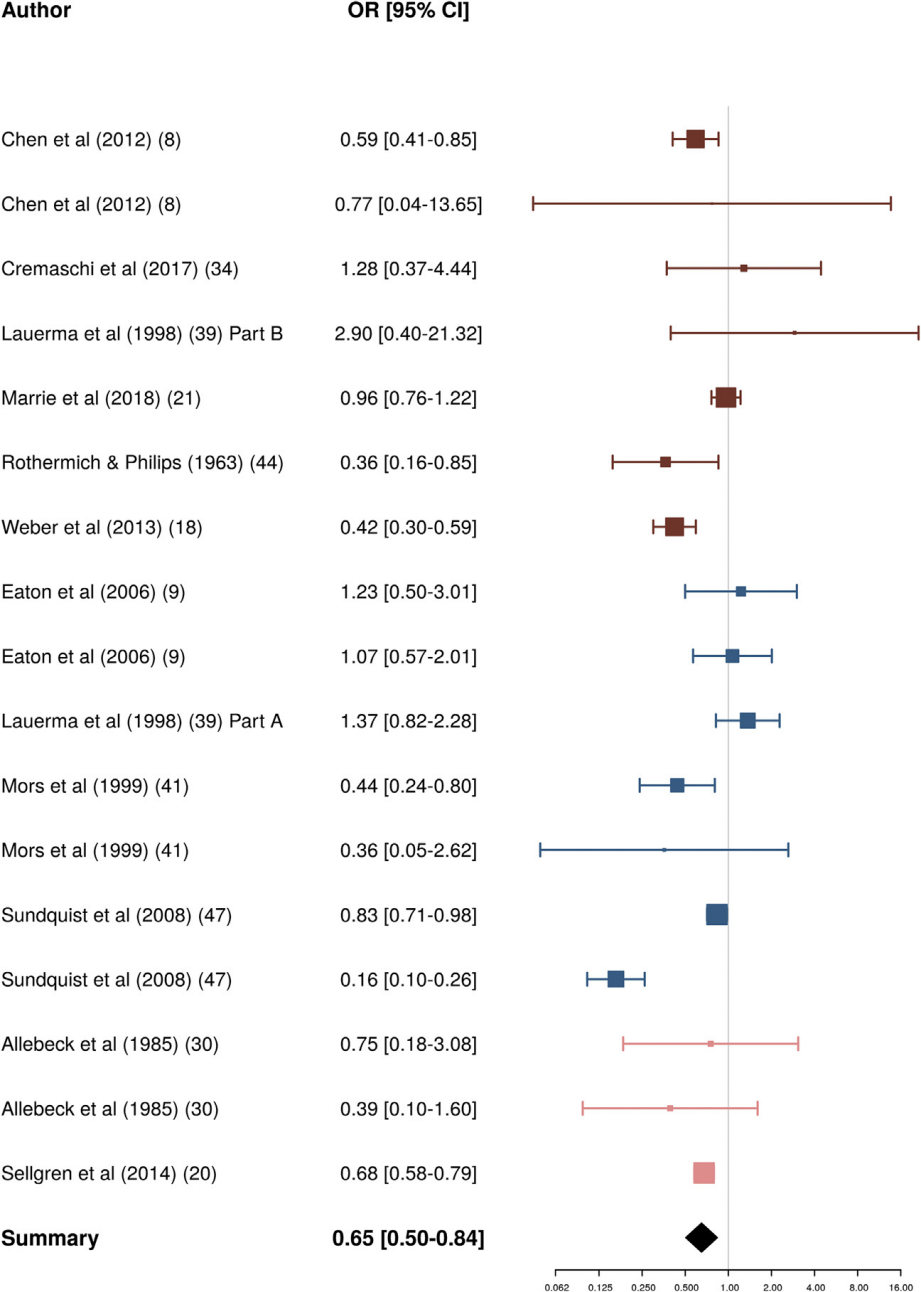
Results of meta-analysis for rheumatoid arthritis and psychosis. All studies examine schizophrenia with the following exceptions: Allebeck *et al.* (1985; second row) (30), nonschizophrenia psychosis; Sundquist *et al.* (2008; first row) (47), nonschizophrenia psychosis; Rothermich and Philips (1963) (44), broadly defined psychosis. Specific rheumatoid arthritis subtypes are as follows: Chen *et al.* (2012; second row) (8), juvenile-onset; Eaton *et al.* (2006; second row) (9), seropositive; Mors *et al.* (1999; second row) (41), juvenile-onset. Marker and line colors indicate study design: type A (red), type B (blue), type C (pink). CI, confidence interval; OR, odds ratio.

Separate meta-analyses were conducted for individual autoimmune disorders where more than three effect sizes were available for analysis (Table 2). A significant positive association was observed for pernicious anemia (OR = 1.91; 95% CI, 1.29–2.84), pemphigoid (OR = 1.90; 95% CI, 1.62–2.24), psoriasis (OR = 1.70; 95% CI, 1.51–1.91), celiac disease (OR = 1.53; 95% CI, 1.12–2.10), and Graves’ disease (OR = 1.33; 95% CI, 1.03–1.72). Significant negative associations with psychosis were observed for both ankylosing spondylitis (OR = 0.72; 95% CI, 0.54–0.98) and rheumatoid arthritis (OR = 0.65; 95% CI, 0.50–0.84) (Figure 3). No significant associations with psychosis were observed for alopecia areata, Crohn’s disease, polymyalgia rheumatica, SLE, type 1 diabetes, or ulcerative colitis. Of the seven autoimmune disorders significantly associated with psychosis, heterogeneity estimates were possibly unimportant to moderate, and not statistically significant, for pernicious anaemia, ankylosing spondylitis, celiac disease, Graves’ disease, and pemphigoid, whereas significant, moderate to substantial heterogeneity was detected for psoriasis and rheumatoid arthritis (Table 2). Visual inspection of the funnel plot for rheumatoid arthritis indicated no substantial asymmetry (Supplemental Figure S1B).

## Discussion

This is the first meta-analysis to examine the association between multiple NNAI disorders and psychosis. Our primary analysis (which excluded rheumatoid arthritis) showed evidence of a generic positive association between NNAI disorders and psychosis. While the overall effect size was small (OR = 1.26), and substantial heterogeneity was detected, this positive association was consistent across study designs and psychiatric outcomes. Analyses conducted for separate NNAI disorders showed significant positive associations for pernicious anemia, pemphigoid, psoriasis, celiac disease, and Graves’ disease and significant negative associations for ankylosing spondylitis and rheumatoid arthritis.

Stratified analyses demonstrated that not only is there increased comorbidity between NNAI disorders and psychosis (type A), but also NNAI disorders increase the risk for subsequent psychosis (type B) and vice versa (type C). Similarly, the positive association we observed was consistent across psychiatric diagnoses despite the fact that analyses performed in these subgroups were likely underpowered. However, heterogeneity was not improved when we stratified by these variables, which likely reflects the wide range of NNAI disorders examined. Stratification by NNAI disorder improved heterogeneity estimates for some conditions but not others (alopecia areata, Crohn’s disease, psoriasis, rheumatoid arthritis, SLE, and type 1 diabetes). Study factors (e.g., country and data source) and participant factors (sex and treatment) may contribute to the residual heterogeneity observed within these NNAI disorders.

Our analyses were restricted to studies that provided raw data that could be used to calculate ORs, thereby precluding us from including data from two nationwide studies, each examining multiple NNAI disorders, that specifically addressed temporal effects 7, 23. Benros *et al.* (7) reported that the presence of any prior autoimmune disorder increased the risk of schizophrenia by 1.29-fold, whereas schizophrenia increased the risk for subsequent autoimmune disorder by 1.53-fold (23). As these results are consistent with the summary ORs that we derived from type B (OR = 1.43) and type C (OR = 1.55) studies, it is unlikely that these data would have altered the significant positive associations that we observed, although the statistical significance of our type C analyses (which were likely underpowered) may have increased.

Multiple factors have been suggested to underlie the observed association between NNAI disorders and psychosis, including inflammation, shared genetic vulnerability, predisposing infections, and brain-reactive antibodies (10). Several lines of evidence support the inflammatory hypothesis of psychosis. First, elevated levels of inflammatory markers (i.e., C-reactive protein and cytokines) and proinflammatory cells (e.g., T helper 17 cells) have been observed among individuals with schizophrenia 52, 53, 54, 55, 56, 57. Second, increased activity of the complement system has been reported in both schizophrenia (58) and autoimmune disorders (59). Finally, proinflammatory cytokines have been associated with smaller hippocampal volume in patients with first-episode psychosis (60) and shown to predict progressive thinning of the prefrontal cortex among individuals at clinical high risk for psychosis (i.e., individuals thought to be in the putatively prodromal phase of illness based on their clinical presentation), which was in turn associated with transition to psychosis (61). Although activation of the immune system is a core feature of all autoimmune disorders, differences in the downstream molecular immune pathways activated across the different autoimmune diseases may partly explain why we observed significant associations for some, but not all, NNAI disorders in stratified analyses.

That there might be a shared genetic link between autoimmune disorders and psychosis is supported by genome-wide association studies showing that immune regulatory genes are significantly associated with schizophrenia (62). Of particular relevance is the human leukocyte antigen (HLA) gene loci, which encode molecules involved in antigen presentation, inflammation, the complement system, and immune responses (63) and have been associated with schizophrenia in numerous genome-wide association studies (64). However, two recent genome-wide association studies failed to show that single nucleotide polymorphisms associated with autoimmune disorders (including ankylosing spondylitis, celiac disease, Graves’ disease, psoriasis, and rheumatoid arthritis) were enriched in schizophrenia 65, 66. In contrast, recent studies have reported a significant negative single nucleotide polymorphism–genetic correlation between schizophrenia and seropositive cases of rheumatoid arthritis (67) and have identified single nucleotide polymorphisms with potential pleiotropic effects for schizophrenia and rheumatoid arthritis (i.e., where allelic variants of the same gene increase the risk for different disorders) (68). Thus, shared genes (particularly HLA genes) might explain the negative associations we observed between psychosis and rheumatoid arthritis, but not the positive associations we found with other NNAI disorders or the negative association with ankylosing spondylitis.

Infectious diseases are thought to play a role in the etiology of autoimmune disorders (69). A recent study from Denmark indicated that severe infections resulting in hospitalization (including bacterial, viral, and other causes of infection) increase the risk for many autoimmune disorders, including anemia, celiac disease, pemphigoid, psoriasis, and rheumatoid arthritis (70). Moreover, data from the same population show that the risk of schizophrenia is even higher among individuals exposed to both an autoimmune disorder and serious infection (7). Thus, prior infection could increase the risk for both NNAI disorders and psychosis. However, evidence regarding the role of specific pathogens in NNAI disorders is often lacking, and it is possible that HLA genes [which have been associated with risk of developing both infections and autoimmune diseases (63)] might explain these associations. Moreover, infection is associated with elevated risk of ankylosing spondylitis and rheumatoid arthritis (70), which in the current study were negatively associated with psychosis. Thus, infection is unlikely to fully explain the observed pattern of results.

There is current interest in the role of neuronal surface autoantibodies in psychosis (71). These antibodies (most of which have been characterized only recently) can induce autoimmune encephalopathies, in which psychotic symptoms are frequently featured. Antibodies directed against the anti–*N*-methyl-D-aspartate receptor are of particular interest given the links between this receptor and psychosis, with a recent meta-analysis finding that these neuronal surface autoantibodies are more commonly detected among individuals with psychosis relative to healthy control subjects (72). Given that studies of neuronal surface autoantibodies are in their infancy, the extent to which they might explain the association between autoimmune disorders and psychosis is unclear, particularly because no studies to date have examined the prevalence of these antibodies in individuals with NNAI disorders. However, encephalopathy associated with autoimmune thyroid disease (also known as Hashimoto’s encephalopathy or steroid-responsive encephalopathy associated with autoimmune thyroid disease), a condition associated with neurological and psychiatric symptoms, has been observed among individuals with Graves’ disease, all of whom presented with antithyroid antibodies (73). Moreover, a recent study reported that antithyroid antibodies were present in 13% of patients with schizophreniform disorder (74). Further research is needed to determine the extent to which specific autoantibodies might mediate the association between the specific NNAI disorders and psychosis that we observed.

The potential contribution of corticosteroid treatments to the association between NNAI disorders and psychosis has received relatively little attention. This is surprising given that there is robust evidence of glucocorticoid (i.e., cortisol) abnormalities among individuals with, and at risk for, psychosis (75). Moreover, a recent population-based study reported increased risks for schizophrenia spectrum disorders among children and adolescents who received glucocorticoid treatment (76). Of particular relevance to our findings, corticosteroids are among the most common types of treatment for psoriasis and pemphigoid 77, 78, 79 and some forms of autoimmune anemia (80). However, corticosteroids are also commonly used in the treatment of rheumatoid arthritis (81), which was negatively associated with psychosis. Thus, the contribution of corticosteroids to these associations is currently unclear.

The negative association we observed between rheumatoid arthritis and psychosis is consistent with an earlier meta-analysis (82). Given the late age of onset for rheumatoid arthritis, it possible that this relationship is partly explained by reduced life expectancy and poorer health care (leading to lower detection rates) among people with psychosis. Of note, rates of cancer, a disease that typically has its onset in later life, are also lower among individuals with psychosis (83). Consistent with this explanation, juvenile-onset rheumatoid arthritis was not significantly associated with psychosis (Figure 3), although juvenile-onset and late-onset forms differ with regard to symptom severity and treatment (84). Moreover, the fact that significant negative associations were found across type A 8, 18, 44, B 41, 47, and C (20) studies is at odds with this hypothesis and instead suggests that a third factor (e.g., genetic influences or treatment) may underlie this association.

One novel finding was the significant negative association observed between ankylosing spondylitis and psychosis. While this result is perhaps unsurprising given that ankylosing spondylitis was initially thought to be a type of rheumatoid arthritis, the etiology and presentation of these conditions differ substantially (85). Specifically, the age of onset is far earlier for ankylosing spondylitis than for rheumatoid arthritis; the former is more common in male patients, whereas the reverse is true for rheumatoid arthritis; and the disorders are associated with different HLA genes (86). Further investigation is needed to identify factors (including other polygenes) common to both disorders that might explain their negative association with psychosis. As a related issue, it is interesting to note that we observed positive associations with NNAI disorders that are traditionally classified as organ-specific (pernicious anemia, Graves’ disease, pemphigoid, and psoriasis) but negative associations with systemic NNAI disorders (ankylosing spondylitis and rheumatoid arthritis) that target multiple organs. However, the distinction between organ-specific and systemic disorders is not clear cut, and current categorization is largely based on clinical presentation as opposed to the expression pattern of the self-antigen (87).

While psychotic symptoms are a common neuropsychiatric feature of SLE (88), SLE was not significantly associated with psychosis in this meta-analysis; in fact, only one study meeting inclusion criteria reported a significant positive association for SLE and psychosis (48). This may be because psychoses or psychotic manifestations in SLE can be acute and transient and therefore not equivalent to a diagnosis of psychotic disorder. In addition, the studies that do refer to diagnosed psychotic disorders in SLE are often isolated case reports (89) or studies that do not include control groups (90), which were not examined in this meta-analysis.

### Strengths and Limitations

The vast quantity of data examined (>25 million individuals) is a major strength of this study. Our search strategy was conducted to ensure that we captured all relevant studies, including some very early publications. A further strength relates to the fact that we systematically investigated the effect of temporality, psychiatric diagnosis, and specific NNAI disorders on the magnitude and consistency of effects.

Some limitations must be noted. First, owing to substantial variation in the effect size measures reported across studies, we extracted raw data to compute ORs. This meant that our effect sizes were not adjusted for important confounding factors that may have influenced the association. However, half of the included studies matched cases and controls on age and sex (Table 1). Second, as we were keen to use all available data, we included studies using both small clinical samples and large nationwide populations, which may have contributed to heterogeneity. Third, as this study was undertaken for partial fulfillment of a Master’s thesis (SH), a protocol was not published before undertaking the study, and as such our analysis strategy may have been driven by the data. Fourth, by extracting (and pooling) data for individual NNAI disorders, patients with more than one NNAI disorder may have been counted twice in the primary (overall) analysis. Fifth, we examined a wide range of disorders classified as autoimmune in the primary articles that included some diseases for which an autoimmune basis has not yet been demonstrated. However, to increase validity, we included only NNAI disorders listed as such by the American Autoimmune and Related Diseases Association. Finally, despite efforts to obtain data from study authors for all eligible studies, we were unable to include data from two large, nationwide studies 7, 23. However, as noted above, it is unlikely that including these data would have substantially altered the overall finding.

### Implications

The finding that psychosis is associated with NNAI disorders (i.e., autoimmune disorders that would not be expected to directly target the brain but nonetheless generate substantial immune system activation in the peripheral systems that might ultimately affect the brain) is particularly important. However, given the range of possible mechanisms that may underlie the significant associations that we observed, the considerable heterogeneity across studies, and the fact that all effect sizes were small, treatment recommendations based on these findings would be premature. Regardless of the mechanism, these findings suggest that careful monitoring of individuals with specific autoimmune diseases (particularly anemia, Graves’ disease, and pemphigoid, as these were the most consistent effects) for early signs of psychosis is warranted.

With regard to future research, we recommend the following: 1) studies should be designed to better disentangle the temporal nature of the association between NNAI disorders and psychosis, as such studies have demonstrated that both psychosis 7, 23 and depression (91) show bidirectional associations with autoimmune disorders; 2) larger studies should be undertaken to examine the presence of neuronal surface autoantibodies among individuals with psychosis; 3) greater efforts should be made in large cohort studies to include information on potential confounders, such as socioeconomic status, adversity, and tobacco use; and 4) studies should be undertaken to evaluate the effect of corticosteroid treatment on the relationship between NNAI disorders and psychosis.
