# Supplementary material for: Associations Between Non-neurological Autoimmune Disorders and Psychosis: A Meta-analysis
Source: Biol Psychiatry. 2019 Jan 1;85(1):35–48. doi: 10.1016/j.biopsych.2018.06.016 (PMC6269125; doi:10.1016/j.biopsych.2018.06.016)
Supplement: Supplemental Material [file mmc1.pdf]

ASSOCIATIONS BETWEEN NON-NEUROLOGICAL AUTOIMMUNE DISORDERS AND PSYCHOSIS: A META-ANALYSIS

SUPPLEMENTARY INFORMATION

Checklist for Meta-Analyses of Observational Studies in Epidemiology (MOOSE) (1)

Supplementary Table S1. MOOSE Checklist Items

Item	Location in manuscript and comments
· Problem definition	Introduction, para 1.
· Hypothesis statement	Introduction, para 2.
· Description of study outcome(s)	Data Extraction, para 1.
· Type of exposure or intervention used	Data Extraction, para 3.
· Type of study designs used	Data Extraction, para 4.
· Study population	Not applicable.
· Qualifications of searchers (e.g., librarians and investigators)	The search terms were selected by the first (A.E.C., PhD) and second author (S.H., MSc). Searches were conducted by S.H. under the guidance of A.E.C., due to the large number of studies identified in the initial search, it was not possible for two reviewers to complete the search independently. However, after excluding articles that were clearly not relevant (based on the title and abstract), two of us (A.E.C. and S.H.) reviewed the full text of all potentially eligible studies to determine inclusion. Disagreements were resolved by discussion with all study authors.
· Search strategy, including time period included in the synthesis and keywords	Search Strategy
· Effort to include all available studies, including contact with authors	Where full text articles were not publicly available, study authors (first and/or senior author) were contacted on at

Item	Location in manuscript and comments
	least two occasions to obtain the full text (Search Strategy).
· Databases and registries searched	Search Strategy
· Search software used, name and version, including special features used (e.g., explosion)	Search Strategy
· Use of hand searching (e.g., reference lists of obtained articles)	Reference lists of all review articles and commentaries identified in the initial search were manually searched to identify further eligible studies (Search Strategy).
· List of citations located and those excluded, including justification	Due to the large number of citations identified (N=6,307) a full list of the articles identified in the initial search is not provided. The total number of papers that were identified, excluded (with justification provided), and included in the final analysis are presented in Figure 1. Citations for all papers included in the meta-analysis are provided in the manuscript (Search Results).
· Method for addressing articles published in languages other than English	Whilst a substantial number of non-English studies were identified in the initial search, a review of the abstract and title indicated that none of these articles were eligible for inclusion; thus, translation of the full text was not necessary.
· Method of handling abstracts and unpublished studies	Search Results
· Description of any contact with authors	Data Extraction, para 3.
· Description of relevance or appropriateness of studies assembled for assessing the hypothesis to be tested	Data Extraction, para 1.
· Rationale for the selection and coding of data (e.g., sound clinical principles or convenience)	Data Extraction, para 2 & 3.
· Documentation of how data were classified and coded (e.g., multiple raters, blinding, and interrater reliability)	Data Extraction, para 1, 2 & 3.
· Assessment of confounding (e.g., comparability of cases and controls in studies where appropriate)	Data Extraction, para 2.
· Assessment of study quality, including blinding of quality assessors; stratification or regression on possible predictors of study results	Description of study quality rating (Data Extraction, para 4); quality rating criteria (Supplementary Table 3) ratings for each study (Table 1).
· Assessment of heterogeneity	Statistical Analyses, para 2.

Item	Location in manuscript and comments
· Description of statistical methods (e.g., complete description of fixed or random effects models, justification of whether the chosen models account for predictors of study results, dose-response models, or cumulative meta-analysis) in sufficient detail to be replicated	Statistical analyses, para 1.
· Provision of appropriate tables and graphics	Figures 1, 2 and 3, Tables 1, and 2, and Supplementary Material.
· Graphic summarising individual study estimates and overall estimate	Figures 2 and 3.
· Table giving descriptive information for each study included	Table 1.
· Results of sensitivity testing (e.g., subgroup analysis)	Results section and Table 2.
· Indication of statistical uncertainty of findings	Table 2.
· Quantitative assessment of bias (e.g., publication bias)	Justification for not conducting quantitative assessment (Statistical Analysis, para 2), graphical presentation (funnel plots) provided in Supplementary Material.
· Justification for exclusion (e.g., exclusion of non–English-language citations)	NA
· Assessment of quality of included studies	Table 1.
· Consideration of alternative explanations for observed results	Discussion, para 3-12.
· Generalization of the conclusions (i.e., appropriate for the data presented and within the domain of the literature review)	Implications.
· Guidelines for future research	Implications.
· Disclosure of funding source	Funding.

Search Terms for Individual Non-Neurological Autoimmune Disorders

Searches included the terms “psychosis” or “schizophrenia” or “non-affective psychosis” or “clinical psychotic symptoms” combined with “autoimmune disorders” or “autoimmune diseases” or “XXX”, the latter representing each of the 37 non-neurological autoimmune disorders listed in Supplementary Table S2.

Supplementary Table S2. Search Terms used to Identify Non-Neurological Autoimmune Disorders

Disorder
• Addison disease
• Alopecia areata
• Ankylosing spondylitis
• Autoimmune hepatitis or chronic active hepatitis or primary biliary cirrhosis
• Autoimmune thyroiditis, thyrotoxicosis or hyperthyroidism or hypothyroidism or thyroid disorder or Graves’ disease
• Behcet’s disease
• Celiac or coeliac disease
• Crohn’s disease
• Dermatomyositis
• Endometriosis
• Goodpasture syndrome
• Hereditary haemolytic anaemia
• Hypersensitivity vasculitis
• Idiopathic thrombocytopenic purpura
• Interstitial cystitis
• Juvenile arthritis or juvenile idiopathic arthritis or arthritis or rheumatoid arthritis or seropositive rheumatoid arthritis or seronegative arthritis
• Kawasaki disease
• Mixed connective tissue disease
• Myositis
• Pemphigoid
• Pemphigus vulgaris
• Pernicious anaemia
• Polymyalgia rheumatica
• Primary adrenocortical disease
• Primary sclerosing cholangitis
• Psoriasis or psoriasis vulgaris
• Psoriatic arthritis
• Purpura
• Sarcoidosis
• Sjorgen’s syndrome
• Systemic lupus erythematosus
• Systemic sclerosis or scleroderma
• Type 1 diabetes or diabetes type 1 or insulin dependent diabetes or diabetes mellitus
• Ulcerative colitis
• Uveitis or iridocyclitis
• Vitiligo
• Wegener granulomatosis

Inclusion and Exclusion Criteria

Supplementary Table S3. Inclusion and Exclusion Criteria for Studies

Inclusion Criteria

1. The presence of schizophrenia or a psychotic disorder (including both affective and non-affective psychosis).
2. Examines one or more non-neurological autoimmune disorders, including disorders which can affect the central or peripheral nervous systems (e.g., systemic lupus erythematosus and Sjorgen's syndrome) but which are not classified as neurological conditions.
3. Inclusion of a healthy control group (i.e., control groups comprising individuals with other, non-psychotic psychiatric disorders were not eligible).
4. Report includes sufficient data to allow computation of an odds ratio (OR).

Exclusion Criteria

1. Autoimmune disorders detected using antibodies only.
2. Only genetic overlap between psychosis and autoimmune disorders was examined.
3. The sample overlapped (and was smaller) than another eligible study.
4. The disorder examined was not sufficiently specific to disambiguate autoimmune and non-autoimmune types (e.g., 'diabetes').

Quality Rating for Included Studies

All studies were rated for methodological quality using a modified version of the Newcastle-Ottawa Scale (2). Criteria for each of the three study designs (cross-sectional, case-control, and cohort) were amended to allow a maximum total score of eight for each study.

Supplementary Table S4. Scoring for Quality Rating of Studies

Criteria	Max score
**Cross-sectional studies**	
· Sample representative of target sample (e.g., all eligible or random sample)?	1
· Sample size justified and satisfactory?	1
· Non-response rate is defined, satisfactory, and characteristics of responders/non-responders compared?	1
· Ascertainment of exposure is valid and/or well-described?	1
· Participants in different outcome groups are comparable and confounders adjusted for?	2
· Assessment of outcome is robust with diagnostic tool and/or record linkage?	1
· Outcome per group reported appropriately?	1
**Case-control studies**	
· Case definition adequate (e.g., independent validation with tool or record linkage demonstrates that diagnoses accurate)?	1
· Cases are representative (i.e., all cases or randomly selected)	1
· Selection of controls (are they representative)?	1
· Definition of controls (steps taken to confirm do not have the outcome of interest?)	1
· Cases and controls are matched on at least one factor?	1
· Exposure ascertainment is robust, uses diagnostic tool, not based on self-report, collected blindly?	1
· Same method of ascertaining exposure in cases and controls?	1
· Non-response rates are reported and same in case and controls?	1
**Cohort studies**	
· Representativeness of exposed cohort (e.g., total pop or random sample, selected group)	1
· Non-exposed group is selected from same population as exposed?	1
· Method used to ascertain exposure is robust?	1
· Steps taken to confirm outcome not present at the start?	1
· Exposed and unexposed are matched or adjustment for confounding occurs?	1
· Assessment of outcome was blind to exposure status or used record linkage, were diagnostic tools used?	1
· Follow-up period was sufficiently long for outcomes to occur?	1
· Loss to follow-up rate is reported, low (<30%), and same in exposed and non-exposed?	1

Funnel Plots to Assess Small Sample Bias

Panel A: Funnel Plot for All Non-Neurological Autoimmune Disorders Included in Primary Analysis

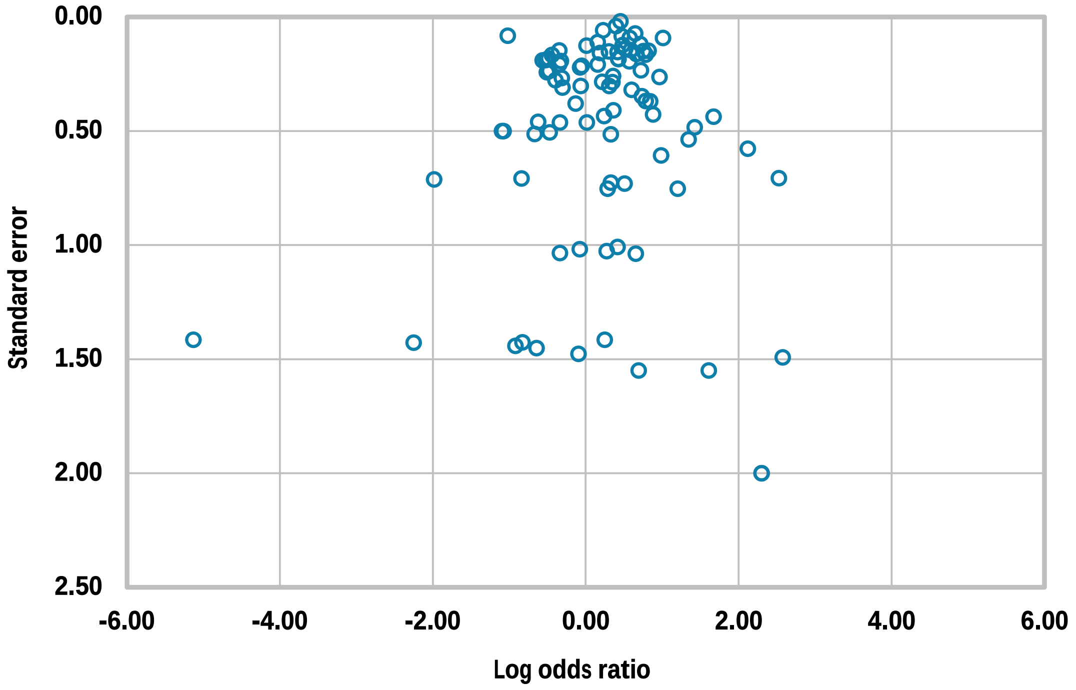

Panel B: Funnel Plot for Rheumatoid Arthritis

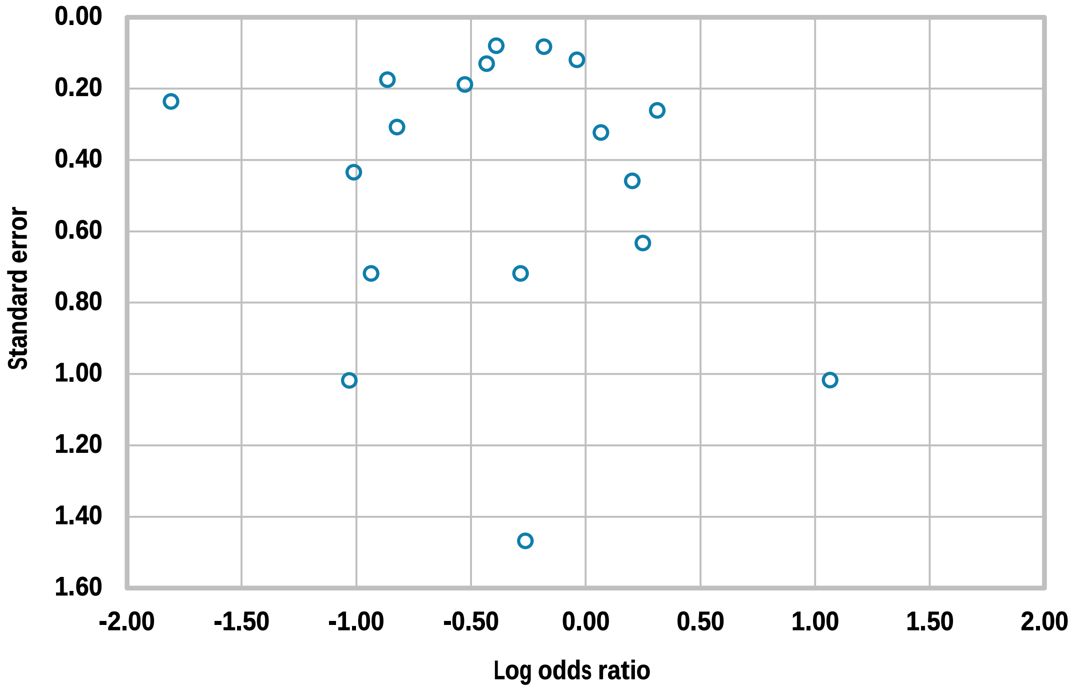

Supplementary Figure S1. Funnel Plots Produced for Analyses With 10 or More Effect Sizes

Supplemental References

1. Stroup DF, Berlin JA, Morton SC, Olkin I, Williamson GD, Rennie D, et al. (2000): Meta-analysis of observational studies in epidemiology: a proposal for reporting. Meta-analysis Of Observational Studies in Epidemiology (MOOSE) group. JAMA. 283:2008-2012.
2. Wells GA, Shea B, O'Connell D, Peterson J, Welch V, Losos M, et al. (2011): The Newcastle-Ottawa Scale (NOS) for assessing the quality of nonrandomised studies in meta-analyses.
